# Changing mega-events’ spatial strategies and cultural policy: scaling down, spacing out, and assembling organizations in the cases of London and Milan

**DOI:** 10.1186/s40410-022-00175-0

**Published:** 2022-11-04

**Authors:** Zachary M. Jones, Stefano Di Vita, Davide Ponzini

**Affiliations:** DASTU - Politecnico di Milano, Milan, Italy

**Keywords:** Mega-events, Cultural Olympiad, Uncertainty, Spatial policy, Cultural policy, London 2012 Olympics, Milan 2015 Expo, Milano-Cortina 2026 Olympics

## Abstract

Despite the proposed ‘certainty’ in a city or region hosting a mega-event, there has long been issues of uncertainty surrounding the planning and implementation of what have until now essentially been mega-projects. Large events have found a variety of ways to adapt and respond to unforeseen circumstances due to political conflicts, planning of oversize venues, limited time of implementation, and legacies that are difficult to manage. Considering the further increased uncertainty surrounding the planning of mega-events as a result of the COVID-19 pandemic, this paper examines how an accompanying cultural component—e.g., the Cultural Olympiad—may help cities that plan for events like the Olympics transition toward diversified drivers and long-term legacy. In particular, the case of cultural offering in Milan between the two mega-events of Expo 2015 and the upcoming 2026 Milano-Cortina Olympic Games shows how host cities can address growing uncertainty through the expanded role of a cultural programme combined with the rescaling of traditional mega-event formats. With this purpose, 2012 London Cultural Olympiad has been selected as an antecedent example—with positive and negative outcomes—to critically review the relationships between the Olympics and the Cultural Olympiad, as well as their widespread spatial strategy and public engagement. In addition, the paper offers more general conclusions regarding learning potentials of jointly studying mega-events and cultural policy.

## Introduction

Mega-events are extraordinary but recurrent occasions to study processes of urban change (Roche 2000; Wilson and Huntoon 2001). Even before the changes introduced by the COVID-19 pandemic, mega-events were already shifting trajectory, moving away from infrastructure heavy approaches to ones emphasizing re-use and sustainability (Jones 2020). This new thinking on how cities host mega-events can be most clearly seen in the proposed plans for the upcoming 2024 Paris and 2026 Milano-Cortina Olympic Games that tout the use of existing venues and spaces, reducing new construction as much as possible (Paris 2024 2018; Milano Cortina 2026 2019). This transition from a primarily physical or tangible legacy, usually comprised of new infrastructure and facilities, introduces the possibility for widespread and even intangible legacies, more focused on the new uses of spaces in host cities and/or regions. Accordingly, this paper considers the specific role of cultural events in these new processes. Different events can be considered “mega” in many ways (Müller 2015); strict classifications should not limit the potential learning across types of mega-events, in particular excluding long-standing programmes such as the European Capital of Culture (Ponzini 2022). Cultural events, while by no means immune to the many negative effects of the pandemic, have indeed shown a degree of adaptability and flexibility in light of unforeseen circumstances. Considering the growing uncertainty surrounding the planning of all types of mega-events (from political and planning issues, to financial and pandemic crises), this article examines how an accompanying cultural component—e.g., the Cultural Olympiad—may help cities ‘fill in the gaps’ as events like the Olympics and Expos transition to diverse and multiple urban and digital spaces. Our work derives from this hypothesis of the potentially of programs of the Cultural Olympiad, but does not assume a position of either boosterism or antagonism per se. It critically analyses the intersection of mega-events and cultural policy, with specific attention to the changes induced in the urban and metropolitan space over time.

In order to discuss mega-events across different sizes and types of events (e.g., Olympics, Expo, European Capital of Culture), we adopt a qualitative perspective rather than a strict quantitative classification based on number of visitors, overall cost, media exposure or other parameters. The “Methods” section in this article presents the qualitative methods used in the research, that is based on the analytical framework of *Umbrellas, Incubators, Mothers and Killers* as proposed by Jones and Ponzini (2021). The “Literature” section reviews the state of the art of the current literature on mega-events and their multiple uncertainties. Within the “Results and discussion” section, we present the individual cases of the 2012 London Cultural Olympiad and the 2015 ExpoinCittà programme in light of the analytical framework and a new consideration for the accomplishments of these events. The two case studies are selected according to high investments in and experimentation with the organization of side events by the host cities to the Olympics (London 2012) and the Expo (Milan 2015), which are unprecedented. Based on the observation of the two past cases and the changes ongoing internationally, the conclusions represent a set of reflections concretely connected to the upcoming 2026 Milano-Cortina Cultural Olympiad, and more generally regarding the potential contributions of cultural policy for cities hosting mega-events in this phase of uncertainty.

## Methods

### Key methodological aspects

Cultural events such as the Cultural Olympiad and the side Expo programmes face significant and multifaceted uncertainties, both in relation to mega-event and post-event planning processes, as well as in terms of public participation. Usually, this is visible in relation to the long-term development and mega-event legacy. Accordingly, we first completed a state-of-the-art literature review to explore the question of multiple uncertainties regarding the hosting of mega-events, that until recently has been in part overlooked. This background provides the basis from which to approach the two cases of London (hosting the 2012 Cultural Olympiad) and Milan (evolving cultural activities from the 2015 Expo to the 2026 Winter Olympics). This paper does not aim to compare different case studies. We selected the only existing framework that connects mega-events with accompanying cultural events by discussing four types (*Umbrellas, Incubators, Mothers and Kill*ers). This framework has been selected as it provides an articulated position from which to observe the mega-events and their effects over time with the purpose of deconstructing the spatial arrangements and cultural policy. In particular, we consider the combination of spatial strategies and cultural programmes within the analysed case studies of London and Milan by checking their multiple locations and contributions to the host cities and regions.

The 2012 London Cultural Olympiad has been selected as an often-proclaimed successful example to review more precisely what kind of relationships to expect between the Olympics and the Cultural Olympiad. This event has been perceived as a model and mobilized in other situations, despite its social and political limitations (Moore et al. 2018). This ambivalence in the London example allows for a significant problematization of the framework, rather than a simple model to apply to the Milan case. The research of the 2012 London Cultural Olympiad relied on available secondary sources including existing research reports, event plans, evaluation documents and available online presentations as they provided adequate documentation through which to analyse the event through the lens of the four types.

We then present an analysis of the Milan case study (that is, the Expo 2015 and its accompanying *ExpoinCittà* cultural programme). The same framework has been used to critically analyse the planning of 2026 Milano-Cortina Winter Olympics and the potential learnings for the future. Milan has been selected due to this progression of hosting two separate mega-events, but with clear legacy during the interim period of expanding the city’s events and cultural policy. After the experimentation with widespread cultural side events to the official site of the Milan Expo 2015, the Milan-Cortina 2026 are the first Winter Olympic Games to follow many of the precepts of the 2020 Agenda (IOC 2014) by reducing the overall investment in new infrastructure and venues, as well as dividing the hosting duties across multiple cities and different regions. The potential of the future Milano-Cortina’s Cultural Olympiad is clear, despite the limited planning done so far (Di Vita and Wilson 2021). A similar set of secondary sources as London has been used for the case of Milan. In addition, 11 semi-structured interviews were carried out regarding the concluded Expo 2015 and the upcoming 2026 Olympics. These interviews provided key insights into the link between these two events, and how former experience is informing current culture-based strategies and their potential in relation to the future mega-event. The interviewees include policy makers from the public sector (e.g., City government, event planning agency, superintendency) as well as from the private and non-profit sector (e.g., the Milano-Cortina 2026 Foundation, consultancy company). We also explored additional datasets with information pertaining to the occurrence and location of cultural events from the period of 2017–2020 in the metropolitan area of Milan, from which we conducted a GIS mapping and spatial analysis. These findings are not presented in this paper but served as a background and a way to corroborate the qualitative findings from the policy analysis and interviews.

### Applying the *Umbrellas, Incubators, Mothers *and* Killers* framework

The *Umbrellas, Incubators, Mothers and Killers* analytical framework (Jones and Ponzini 2021) considers four potential types of relationships between a mega-event and the accompanying cultural programme. It is useful for studying the dynamic in hosting a mega-event and the possibilities to grow and improve or stagnate and decline local cultural policy and programming over the long term. In this paper, the four types are used to analyse the example of London and apply key learnings to the case of Milan. The four types are based on a matrix comprised of the decrease or increase of either quality or quantity of events. *Mothers* witness an intense growth in the overall number of events in the period following a mega-event compared to beforehand. On the contrary, *Killers* see an actual decrease compared to the previous situation. These two types depend largely on the availability of legacy funding made available following an event and the commitment to a long-term event strategy. Specifically, in the case of *Killers*, the years-long preparation and budgeting for a single mega-event can drain the financial and expertise resources necessary to continue cultural programmes. Conversely, *Umbrellas* see no significant post-event changes in terms of either quality or quantity, while *Incubator* types could be considered as a specific kind of *Umbrella* where a marked improvement in the quality of existing small and micro events can be observed. While the measurement and evaluation of quality is more challenging to track, these two types tend to depend primarily on the level of capacity built up and retained from the mega-event. These types however do not consider events that imply recent radical uncertainty and shocks which threaten the organisation and implementation of mega-events, such as the Covid-19 pandemic or other threats from war and climate change (which have recently added to the long-term uncertainties due to the acceleration of policy and planning processes in host cities and regions). This analytical framework allows one to clearly highlight the criticalities as well as the potential learnings for Milan and the wider Olympic region in the future. As well, this approach identifies more generally the emerging role for cultural policy to play in not only supporting the Olympic Games but as one of the enduring legacies.

## Literature/state of the art

### Mega-events and the expanding role of cultural policies in uncertain times

Despite the proposed ‘certainty’ in a city or region hosting a mega-event, there have long been issues of uncertainty surrounding the planning and implementation of what have until now essentially been interpreted as mega-projects. In his classic book *Great Planning Disasters*, Hall (1982) discusses the general uncertainty surrounding the planning and implementation of major urban projects. He points out the difficulty or even the impossibility of knowing and predicting the progress of complex processes. In some cases, radical uncertainty is ultimately unavoidable. On one hand, uncertainty can relate to the complex governance systems required to develop such projects while, on the other, can also be fuelled by changing perceptions of reality and values over time. In this context, there are projects that are inevitably destined to be disastrous in terms of cost increases, and of ineffectiveness in producing the expected results or the economic and social effects promised by the proponents.

Specifically in terms of mega-events, forecasts are all too often overly optimistic when compared with the results of past editions, particularly concerning desired economic outcomes. The Major Program Management research group of Oxford University's Saïd Business School, led by Flyvbjerg (2021), has collected systematic data for 19 editions of the Olympic Games since 1960, clearly demonstrating that the Olympics consistently exceed the original predicted costs 100% of the time. In the majority of the cases analysed, the costs are more than double than originally expected. This trend in part results from the "blank check syndrome" to ensure that mega-events are delivered at any and all cost, creating a manufactured or false sense of certainty. Müller (2017) discusses various paradoxes, including the fact that cities and countries of the world compete to host the Olympics while knowing that the benefits are generally overestimated.

In literature there is substantial criticism regarding the problems induced by such mega-event features. Traditionally, uncertainty was framed in these ways, focused on the inability to accurately predict the final effects of major events (costs, revenues, audience) (Jennings 2013). Typically, promoters tend to overestimate the benefits and downplay the difficulties in scheduling complex programmes over several years. Raco (2012, 2014) suggests that governance structures that are functional to the Olympics (with its hard deadlines, great financial costs etc.…) may escape from public control and in fact may serve the elite. After the 2007–2008 financial and economic crisis, Lauermann and Vogelpohl (2017) argue that political contestation against mega-event bids proved to be increasingly effective and this lowers the incentives for cities to bid and face growing political uncertainty.

After the social protests against mega-events in Brazil (Vainer 2016) and due to the pandemic, several factors have been exacerbated by particular uncertainty. For example, the Tokyo 2020 Olympics data reveal that costs more than doubled compared to forecasts, in part as a result of the one-year delay of hosting the Games (McCarthy 2021). The economic woes were only exacerbated by having zero audience in attendance, and thus no tickets purchased or additional revenues raised, as a result of restrictions linked to the COVID-19 pandemic. Yet the extreme condition of the pandemic has confirmed that the mega-event will be carried out as planned and that strategies must be able to adopt to a range of unpredictable situations. In this light, the emerging approaches focused on re-use and adaptation rather than primarily on the construction of new venues appears more sustainable not just because it can lower costs and reduce waste, but also because it can better respond to ever increasing uncertainties. Mega-events such as the European Capital of Culture and the Cultural Olympiad are potentially adaptable and capable of responding to and managing multiple uncertainties due to their activities being not entirely dependent on specific venue requirements (Ponzini et al. 2020). Thus, they can be held in a variety of locations and more easily reused in the post-event. Individual events within these programmes tend to be of smaller dimensions in terms of attendance and more widespread, both spatially throughout the city and over a longer period of time (Ponzini and Jones 2021).

Once seen as an aspect making these kinds of events seem less spectacular than other mega-events, this may now serve as a strategic advantage in their planning and implementation. Di Vita and Wilson (2021) collect a series of contributions which offer an innovative perspective on events smaller (in size) than the Olympics, the Expo and the FIFA World Cup. They argue that the uncertainty factors that characterize larger events seem less severe in relation to events such as the European Capital of Culture and smaller events. This seems to be true not only for the long-term sustainability of the interventions (economic, environmental, social), but also in terms of opportunities for territorial development. The ability to govern smaller events and to mediate the resulting side effects, however, cannot be taken for granted, given that in many cases these are unique processes. A further challenge concerns the transition to smaller events with a more widespread distribution (ibid.). This approach not only shows advantages in terms of efficiency, sustainability, manageability of contingencies, and political consensus, but tends to reduce the level of socio-economic and spatial uncertainty.

The *Events through the COVID-19 Pandemic: Evidence from Europe* (Sanetra-Szeliga et al. 2021) specifically discusses the current uncertainty facing major cultural events in the phase of the COVID-19 pandemic. For example, the European Capitals of Culture Rijeka 2020 and Galway 2020 turned towards more digital events, while Novi Sad 2021 and Elefsina 2021 chose to instead delay their programmes until 2022 and 2023 respectively. With more time available to orient themselves in an unpredictable situation, the cities of Kaunas 2022 and Esch 2022 were able to reprogram and conceive the events differently, both in terms of distribution in the urban space (physical and online presence), and cultural content. In a phase of uncertainty like this, the report draws attention to local residents as consumers and beneficiaries of mega-events and related cultural policies, rather than tourists, given the instability of flows and presences.

Within this framework, this article aims to investigate the potentials of connecting mega-events and cultural policies: on the one hand, discuss the potential contribution of cultural policies during multiple phases of uncertainty; on the other, to improve future mega-events and their long-term legacies, counterbalancing high cost and infrastructure-heavy approaches. We now turn to a detailed analysis and discussion of the two case studies.

## Results and discussion

### *Umbrellas, Incubators, Mothers* and *Killers* in the London 2012 Cultural Olympiad

An artistic or cultural component has accompanied the Olympic Games in some form for over 100 years (García 2017). However, despite this longevity and notable examples of cities that promoted the Cultural Olympiad such as Turin 2006 Winter Olympic Games and the Sydney 2000 Summer Olympic Games (Pappalepore 2016; Stevenson 1997), few host cities have framed the Cultural Olympiad as a key element in their delivery of the Games. London especially highlighted the Cultural Olympiad within their original bid and expanded the event to a four-year programme. A total of £126.6 million was invested across the four years, both in London and across the UK in an estimated 180,000 activities involving up to 43 million participants. The case of London is therefore a notable example for any future Olympic host city to study and better understand the potential of the Cultural Olympiad programme.

The London 2012 Summer Olympic Games were hosted solely by London, whereas the Cultural Olympiad was carried out across the entire UK—culminating in the London 2012 Festival held in the lead up to the Games themselves. To effectively carry out the programme, regional leaders were appointed to coordinate cultural activities in their region during the 4-year period leading up to the Games. A key element in this approach was the unified branding scheme that helped to connect a quite wide-ranging programme (Garcia 2012). This clear imaging linked to the iconic 2012 logo helped to bring recognition to and knowledge of the Cultural Olympiad which was not previously as well understood. In addition, there is a stronger socio-cultural interpretation of the role of such set of events. They can serve in generating a sense of unity and belonging in very different places that may not be directly connected (e.g., through physical infrastructure) to the Olympic venues. However, some issues of disconnection emerged as individual activities were managed quite separately one from another (Gilmore 2010).

London’s approach to spreading the cultural programme at a national scale is not entirely unique but rather reflects to a degree the approach of several city/region hosts of the European Capital of Culture (ECoC). Notably, Essen for the Ruhr 2010 ECoC and the Matera-Basilicata 2019 ECoC both took a regional approach to hosting their year of celebration by spreading events across a wider region—bringing greater attention to often overlooked areas and benefitting the overall programme through using existing resources. However, it is worth highlighting the experience of London and other host cities where the Olympic Games actually kept away visitors looking to avoid higher costs and complications from the Games, leading to a reduced number of tourists during the mega-event itself (Vlachos 2016). As a counter point, the Cultural Olympiad can instead serve to attract visitors in the period leading up to the Games themselves to areas they might not otherwise visit. In this way, it helps to spread the benefits of the Games across a wider region without over-burdening any one single place. The following sections will examine different aspects of the London 2012 Cultural Olympiad through the lens of the four types to reflect upon the varying effects and impacts which at times coalesced and aligned, while in other ways diverged with negative consequences.

#### London 2012 Cultural Olympiad as a spreading *Mother *with and *Incubator*

As sport and cultural mega-events increasingly come to use existing spaces and venues in cities or across regions as opposed to strictly constructing new facilities and infrastructure, there is the greater potential for the use of urban and historic spaces. While the London 2012 Olympic Games was primarily implemented as an anchored platform in East London, made up of newly created venues and spaces, the 2012 London Cultural Olympiad took great efforts to use existing widespread spaces, from the alternative to the iconic as well as the historic. In this way, the Cultural Olympiad was used to develop not only new events, but also spread those events across the city and region in new locations. The *One Extraordinary Day* event saw unique dance performances taking place across the city of London in iconic locations like the Millennium Bridge and City Hall building, both designed by Foster + Partners. These performances were suspended from the structures themselves, turning them into an active component rather than just a static backdrop. Olympic cities have long used the Games to show dramatic images of the city for promotion, and these cultural events can use sites not adapted for sporting venues while yet activating them in new and innovative ways. Beyond London, several historic sites such as Stonehenge and Hadrian’s Wall amongst others served as cultural event locations (García and Cox 2013).

The 2012 London Cultural Olympiad also developed an entire strain of events located in so-called ‘unusual places’ focused on new or innovative event locations. While these included the iconic ones mentioned above, many lesser known or otherwise overlooked spaces also were activated within this programme.

Most sport mega-events have long struggled or not bothered to include public participation processes in meaningful ways in the bidding or planning phases (French and Disher 1997; Haxton 2000; Lenskyj 1992). As an *Incubator*, the cultural programme was highlighted as one of the main elements to benefit a wide range of parties and local communities within the original bid, with diversity and inclusion a key aspect to this approach (García and Cox 2013). The quality of events was improved through this more open approach that embraced both grassroot groups as well as internationally recognized artists. In particular, artists who are deaf and with other disabilities were significantly featured and noted as an important step in making arts and cultural policy more inclusive and accessible (Abrams and Parker-Starbuck 2013). Yet Abrams and Parker-Starbuck also question the relevance of the cultural programme to supporting the local communities negatively impacted by the Olympic Games, particularly those in East London who were relocated to make way for the construction of new venues (ibid.). As with many cultural mega-events, there was also the debate between the building up of local cultural resources versus the displaying of invited international talent along with the divide between so called ‘high’ and ‘low’ artforms (Pappalepore 2016).

#### London 2012 Cultural Olympiad as a medium-term *Killer*

A critical aspect of the 2012 London Cultural Olympiad was the focus on legacy within the event governance. A common shortcoming in mega-event governance is a lack of learning from the process and retaining the capacity building that has occurred (Jones 2022). Within the LOCOG (London Organising Committee for the Olympic and Paralympic Games) there was a Cultural Olympiad Board and specific Culture Delivery Team. These were supported by 13 additional programmers working in the regions across the UK along with several existing entities serving as delivery partners as well as public funding bodies. On one hand, this extensive governance network was needed for the proper delivery of the event programme while, on the other hand, it served to establish greater connections across the cultural sector to endure beyond the 2012 event (Garcia 2012). The post-event report confirms that nearly half of the cultural activities that took place would not have occurred, at least not in their final form, had the Cultural Olympiad not taken place (García and Cox 2013). At first glance this might suggest classifying the Cultural Olympiad as a *Mother* type. However, this document also reports that only half of the programmed events intended to continue beyond 2012, making it difficult to decipher how many events newly generated by the Cultural Olympiad extended to the medium- and long-term following the conclusion of the 2012 Olympic Games. Most reported the lack of continued funding as one of the significant hurdles to continuing cultural events—the usual problem facing operations following cultural mega-events. Without more precise data it is difficult to determine whether the events that continued post-2012 were those pre-existing ones or newly created ones. However, it is most likely that not only the newly created ones, but also part of the pre-existing events ended following 2012. In this sense the Cultural Olympiad could also be classified, in part and for the medium term, as a *Killer* for cultural events.

Generally speaking, local cultural actors and operators involved claimed to benefit from the presence of the Cultural Olympiad by providing new opportunities, work and networks that they might not otherwise have developed (Pappalepore 2016). Despite legacy being a highly discussed and promoted aspect of the 2012 London Cultural Olympiad, there is not a clear, definite continuation of the programme in terms of ongoing activities or even presence of a governance body. While a legacy paper was produced (Cultural Olympiad Board 2011), the document focuses on establishing an evaluation framework to be carried out following the event rather than a set of policy guidelines or practice to continue post-2012. Instead, the main interpretation of legacy seems to have focused on exporting a London 2012 model to future organisers of the Cultural Olympiad in other countries (McDowell 2015). While this approach has helped to secure a positive reputation for the London 2012 Cultural Olympiad as an exemplar to be followed it does little to ensure the continuation of cultural programmes established in the years leading up to 2012. For sure, one effect of the Cultural Olympiad was improving the governance of two subsequent mega-events in other parts of the UK: the Derry-Londonderry 2013 UK City of Culture and the Glasgow 2014 Commonwealth Games (Cox et al. 2014).

### Mega-events in Milan’s culture: from the 2015 Expo to the 2026 Olympic Games

Prior to hosting the 2015 Expo, Milan had a burgeoning cultural scene that since the 2000s had largely developed through the efforts of individual key players (Ponzini et al. 2014). Despite the richness and potential for the cultural sector in Milan, in the 2000s and early 2010s the city lacked a medium-long term vision shared by multiple actors that could allow the city to achieve a higher level of cultural production. The lack of political leadership in addressing the metropolitan scale and in establishing stronger connections with the periphery and hinterland led actors to organize cultural events and activities independently. Throughout the years, the Fuorisalone Design Week and the Fashion Weeks have grown over time and spread in the metropolitan space, regenerating and giving new identities to several former industrial neighbourhoods of Milan (Armondi and Bruzzese 2017), though without a common strategy. Within these wider processes of cultural development, the Expo 2015 came to play a critical role—particularly in terms of capacity building, as well as spatial strategies and cultural policy (Bolocan Goldstein 2015; Pasqui 2015).

According to the regulations set out by the Bureau International des Expositions (BIE), the main 2015 Expo site was established as a single satellite platform between the Municipalities of Milan and Rho, within the Metropolitan City of Milan, 14 km from the centre. For the event celebration, the problem of physical and intangible disconnection between the Expo site and the rest of the city was addressed in multiple ways, in terms of infrastructure and mobility, as well as communication (Di Vita and Morandi 2018). Accordingly, the ExpoinCittà platform and cultural programme launched by the Municipality and the Chamber of Commerce collected 1015 event locations across the Milan region, that included already existing event venues, alternative sites and open-air spaces. The Milan Municipal Administration’s Sportello Unico Eventi (SUEV) was created in order to ease the process of authorizing and monitoring events. A total of 46,310 events were recorded in 2015, with the main goal being to activate the entire city on the occasion of the Expo and attract attention to the city itself along with the Expo site (Di Vita 2022). A total of 11 million visitors were recorded by *ExpoinCittà*, representing more than half of the total 21.5 million visitors to the Expo itself. Events were held in museums along with industrial sites, libraries, historic farmhouse cascinas and villas, leading to a substantial boost in the guidance of cultural events. Despite this spreading of potential event locations, there was a clear concentration of events, with 45% of the programme taking place in the historic city centre and only 10% occurring outside the municipal area (Di Vita and Ponzini 2020). Unlike the experiences of many other cities hosting mega-events, the close of the Expo did not bring about the end of the ExpoinCittà programme, but rather its continuation and rebranding as YES!Milano. The YES!Milano agency continued to organise thousands of events per year following 2015 up until the pandemic shuttered public events in 2020. Many of these events are organised according to thematic weeks and continue to take place across different but specific city districts (ibid.). In this sense, there is little doubt about the successful role as an *Incubator* of ExpoinCittà.

Cities can often struggle to learn from the experience of hosting a mega-event and retaining the expertise gained (Jones 2019), but the case of the 2015 Milan Expo stands out as an exemplar in this specific regard. In fact, when compared to the problematic legacy of the Expo site itself, which remains yet undeveloped and unused as of the end of 2021, the accompanying cultural programme of the *ExpoinCittà* stands out as one of the immediate successful legacies of the 2015 Expo (Di Vita 2022). It has demonstrated the possibility of a mega-event to activate various resources including not only funding and local expertise, but also the cultural activation of spaces across the city and wider metropolitan region over the long term. One of the key elements in Milan’s approach back in the early 2010s was its development of the innovative open-data collection that encouraged thousands of collateral events to take place across the metropolitan region. This additional programme was not requested nor required by the BIE, and it was not extended to the post-event phase, but it is one of the more innovative elements to be suggested for future host cities, based on the positive experience in the case of Milan (ibid.).

The bidding for and awarding of the 2026 Milano-Cortina Winter Olympic Games confirms an enduring positive perception of the 2015 Expo (Bruzzese and Di Vita 2016), despite various shortcomings and reactions against the event (Casaglia 2016; Basso 2017) and the problematic legacy of the event site (Gaeta and Di Vita 2021). The regional spread of the sporting events, across a distance of 400 km, presents the need for a new thinking in terms of scale, organisation and planning—not only for the main sport events, but also for the Cultural Olympiad programme. The original idea of re-using the existing infrastructure of the 2006 Turin Olympics would have made this spatial system broader (Milano-Torino-Cortina), but it proved to be politically unmanageable in the bidding phase.

Throughout the Milano-Cortina 2026 Bidding Dossier, the local, the regional Alpine and the broader Italian culture play a strategic role in supporting the 2026 Olympic Games and contributing to the overall experience, typically in relation to tourism growth and mentioned alongside heritage, nature, art and fashion. However, only a brief overview of the Cultural Olympiad has been provided until now, envisioning cultural activities taking place exclusively in February and March of 2026 (Milano Cortina 2026 2019 ). According to the analysis of the Bidding Dossier (and still waiting for the definitive Games Delivery Plan and Legacy Plan), only some of Milan’s most visible cultural centres are mentioned (such as the Pinacoteca di Brera, La Scala Theatre and the Triennale) to host a series of events organized around six themes: art/photography; music/opera; theatre; cinema; fashion/design/creativity; food. In this early document, no clear connections were made to the ongoing cultural offerings of the city of Milan or connecting to the legacy of the 2015 Expo in growing the city’s cultural capacity for planning and organising events. The document also fails to specifically note how events might be spread across the vast region involved in hosting the Olympic Games. With the upcoming event now only several years away, it is worth considering in greater detail the potential role of the Cultural Olympiad programme. Specifically, it is worth considering how the Cultural Olympiad programme might be able to respond to local and regional needs within the current period of transition of mega-events like the Olympics, also due to the increased uncertainty introduced by the global pandemic in already uncertain planning processes.

The spatial plans of the Milan Municipality and the Milan Metropolitan City were approved after the awarding of Milano-Cortina 2026 (respectively, in late 2019 and in 2021). However, these visions only implicitly shift from large redevelopments to widespread regeneration and branding of peripheral areas, with goals of socio-spatial equity (Pasqui 2018). At the same time, the Bidding Dossier lacks clear input for spatial visions pertaining to both the urban core and region. More detailed plans may yet be revealed in the Games Delivery Plan and the Games Legacy Plan, both expected only in 2022. While Milan clearly has a successful history of maintaining an ongoing cultural programme, it is worthwhile to examine other examples of the Cultural Olympiad to frame its potential for the Milano-Cortina context in this phase of transition and beyond the 2026 event.

## Conclusions

### Linkages between mega-events and cultural policy, and learning opportunities

The examples of the London 2012 Cultural Olympiad and the Milan 2015 *ExpoinCittà* present several key learnings for future host cities. Within the framework of four types of connections between mega-events and long-term cultural policies, we use the upcoming Milano-Cortina 2026 Winter Olympic Games to make these lessons more concrete, evident, and challenging. In particular, we summarize the arguments according to the spatial structure, long-term vision and public participation dimensions, as well as to further problematization of the four proposed types of relations between mega-events and accompanying cultural events. More general considerations regarding collecting data and learning opportunities conclude the paper. These conclusions intend to contribute to developing a Cultural Olympiad of the 2026 Winter Games in light of both the increased uncertainty and the lack of any existing plans to critically analyse.

## Big space and long time

As noted above, Milan-Cortina 2026 represents a new approach as the first Winter Games to fully embrace the 2020 Olympic Agenda (IOC 2014), with two cities simultaneously sharing hosting duties and spreading events over 400 km. With a greatly reduced dependence upon newly built venues and infrastructure, the Cultural Olympiad presents an opportunity to ‘fill in the gaps’ with a cultural programme (both spatial and intangible) that can expand the reach and impact of the Olympic Games during this phase of mega-event transition. In London, where the Olympic events and venues were primarily clustered within the Queen Elizabeth Olympic Park in the East London, the Cultural Olympiad programme was very much used to spread out the effects and reach a far wider audience, not just across London but across the entire UK. Similarly, in Milan, the *ExpoinCittà* programme has expanded the Expo effects in space (at the scale of the metropolitan region) and in time (in the following years and city agendas). Accordingly, while Milan, Cortina and a handful of other small towns will host the sport events of the 2026 Winter Games, the Cultural Olympiad could be used to accompany this diffused event approach and attract greater attention to these areas across the Alpine region than they would otherwise manage on their own. This widescale diffusion of events, particularly in locations often left out of the spotlight of such mega-events, has been highlighted as one of the positive elements of both the 2012 London Cultural Olympiad programme and the 2015 Milan ExpoinCittà programme.

The London case and its shortcomings also shows that such a strategy requires a long-term, legacy-oriented vision with a robust governance. London had already begun preparing the groundwork for their Cultural Olympiad within the original bid, seven years prior to hosting the Olympic Games, with cultural events being staged four years prior to the opening ceremony. Nonetheless, there was a substantial number of cultural organizations and activities that could not survive the post-event phase. In the case of Milano-Cortina, as of 2022, a cultural programme has not been planned as the city and regions, at the time of writing, are still grappling with the COVID-19 pandemic. However, the current situation not only presents hurdles to overcome, but also opportunities to develop. Digital events can take on a greater importance within a Cultural Olympiad, as well as activating the ‘unusual’, iconic or heritage spaces across the region and country (as seen in London and the UK).

The incremental and unplanned strategy of *ExpoinCittà* (first developed in Milan on the occasion of the 2015 Expo in order to utilize and activate thousands of public and private spaces within the metropolitan region) could be spread out to all the areas involved in hosting the 2026 Games. Nevertheless, such a spatially spread approach would require a stronger governance system involving representatives from multiple areas, as well as it could benefit from a spatial pre-selection of areas and actors to involve, so as to avoid the polarization of disparities between urban cores and peripheries that emerged during the Expo.

As seen in both the London and Milan case studies, this diffused approach can risk becoming quite disconnected from both itself as well as to the related mega-event. Close coordination and planning would be necessary to undertake such an initiative. A wide-spread cultural programme should also strive to build in sustained funding sources (not solely dependent upon the Olympics to achieve a long-term legacy). However, this potential input to the planning of the 2026 Cultural Olympiad in space and time has not been considered yet by the official event governance.

### Uncertain participation along the phases

The wide inclusion of local communities has long been a critical element in bidding and hosting for mega-events such as the Olympics and the ECoC. The public participation within the bidding phase as well as its subsequent monitoring in post-event reports has contributed to making it a requirement for host cities. Using the Cultural Olympiad to include more stakeholders within processes can not only cultivate greater support for the Games themselves but also help ensure that shared decisions are made regarding the use of spaces and the future development of the city. Milan faced such a situation when hosting its last mega-event, the 2015 Expo, both when residents protested the destruction of public park space to construct the water-way aqueduct connecting the Expo site to the city centre as well as when thousands of actors were involved by the *ExpoinCittà* programme of accompanying events through a specific and innovative e-participation digital platform (Di Vita and Morandi 2018). The greater inclusion of residents earlier on in the process could help to avoid such scenarios to begin with. Research into the case of London 2012 also found that it was their involvement in the Cultural Olympiad that many participants remembered due to the possibility to be involved and interact more directly as many would have otherwise had little to no contact with the Olympic Games themselves (Abrams and Parker-Starbuck 2013). Such experiences can in and of themselves form part of the intangible legacy of the event to be recognized beyond just the tangible ones.

### Uncertain relations between mega-events and cultural policies over the medium term

While the *ExpoinCittà* is a clear case of *Incubator*, the case of the London 2012 Cultural Olympiad is more difficult to classify according to one single type (Jones and Ponzini 2021), based on the available secondary data. Considering the massive budget, it developed a significant number of cultural events that would have otherwise not taken place without the mega-event, spreading across the country and using new event locations. One could argue it worked as an *Incubator*, as many of the events within the Cultural Olympiad programme existed prior to and following 2012, impacted to varying degrees by the presence of the mega-event in terms of greater funding, improved governance and greater inclusivity. However, the 2012 Cultural Olympiad could also be classified as a medium-term *Killer*, as nearly half of the overall events ceased to continue following 2012. These varying roles of a single cultural mega-event highlight several issues for future events, such as Milano-Cortina 2026, to be aware of and plan for. In this case, the evolution of *ExpoinCittà to* YES!Milano well demonstrates the potential for institutional legacy of mega-events and maintaining the capacity building that has taken place rather than losing it as often occurs (Jones 2022).

### Mega-event transition and learning opportunities

While organisers of a Cultural Olympiad cannot anticipate all possible issues, this analysis of the accompanying events to the London 2012 and the Milan 2015 case studies by using the above-mentioned four types helps to identify the range of potentials and risks. Any future steps that cities take in organizing the Cultural Olympiad should consider the involvement of multiple stakeholders and communities. Developing broad coalitions early on can help to not just build consensus but to ensure that the programme responds to local needs (Viehoff and Poynter 2015), while also identifying the potentially overlooked or underused spaces that might not normally be used. Any strategies developed must also be careful to avoid the pitfalls of a cultural programme leading to the gentrification of sensitive areas. The intentional spreading out of events across a wider region, rather than the typical concentration in a few central areas, can help to avoid both the issues of over-tourism (or conversely lack of tourism) and disruptive socio-economic shifts that threaten rather than support local communities (Muñoz 2015).

With mega-events like the Olympics turning away from heavy infrastructural investments and embracing a greater diffusion of events (Jones 2020), the Cultural Olympiad could work towards filling in the gaps from creating less physical, infrastructural connections to instead more intangible links and cultural connections. The diffusion of events during the Olympic Games can help to avoid the overcrowding of any single individual area, particularly sensitive natural or built heritage.

A final learning from this investigation worth noting is the valuable role that data plays in being able to study and track cultural phenomenon over time. While a qualitative analysis is necessary for a deeper understanding, more detailed data sets (before, during and after events) would help to provide greater insights into evolution of cultural policy and real effects of hosting a mega-event. The tracking of such data (starting from the level of public administrations) should be an element to include in future evaluations of mega-events. Access to more refined data would also allow for finer grained analysis to better understand the long-term cultural impact of mega-events in contributing to spatial trends in cities and wider regions over time. The case of Milan and the availability of data from the 2015 Expo has been critical in revealing the broader impact of that event on cultural policy, despite its more obvious shortcomings in reusing the Expo site itself. The continued study of these elements in light of the upcoming Milano-Cortina 2026 Winter Olympics can greatly expand upon the learnings of these processes and potentially present Milan as interesting case in terms of legacy of continuing cultural activities following a cultural mega-event.

## Data Availability

All data cited in the article is publicly available and can be found as cited.
